# Microscopic Mass Spectrometry Imaging Reveals the Distribution of Phytochemicals in the Dried Root of *Isatis tinctoria*

**DOI:** 10.3389/fphar.2021.685575

**Published:** 2021-06-29

**Authors:** Li-Xing Nie, Jing Dong, Lie-Yan Huang, Xiu-Yu Qian, Chao-Jie Lian, Shuai Kang, Zhong Dai, Shuang-Cheng Ma

**Affiliations:** ^1^ Chinese Academy of Medical Science and Peking Union Medical College, Beijing, China; ^2^ National Institutes for Food and Drug Control, National Medical Products Administration, Beijing, China; ^3^ Shimadzu China Innovation Center, Beijing, China; ^4^ College of Pharmacy, Hebei University of Chinese Medicine, Shijiazhuang, China

**Keywords:** mass spectrometry imaging, *Isatis tinctoria*, atmospheric pressure–matrix-assisted laser desorption/ionization, ion trap–time-of-flight mass spectrometry, traditional Chinese medicine

## Abstract

The dried root of *Isatis tinctoria* L. (Brassicaceae) is one of the most popular traditional Chinese medicines with well-recognized prevention and treatment effects against viral infections. Above 300 components have been isolated from this herb, but their spatial distribution in the root tissue remains unknown. In recent years, mass spectrometry imaging (MSI) has become a booming technology for capturing the spatial accumulation and localization of molecules in fresh plants, animal, or human tissues. However, few studies were conducted on the dried herbal materials due to the obstacles in cryosectioning. In this study, distribution of phytochemicals in the dried root of *Isatis tinctoria* was revealed by microscopic mass spectrometry imaging, with application of atmospheric pressure–matrix-assisted laser desorption/ionization (AP-MALDI) and ion trap–time-of-flight mass spectrometry (IT-TOF/MS). After optimization of the slice preparation and matrix application, 118 ions were identified without extraction and isolation, and the locations of some metabolites in the dried root of *Isatis tinctoria* were comprehensively visualized for the first time. Combining with partial least square (PLS) regression, samples collected from four habitats were differentiated unambiguously based on their mass spectrometry imaging.

## Introduction

Natural products have always benefited the health care of people worldwide and are used as herbal medicines commonly (He et al., 2020). Among them, traditional Chinese medicine (TCM) has made significant contributions to the treatment of human disease from ancient times to present (Wang et al., 2021). For instance, the dried root of *Isatis tinctoria* L. (*Isatis indigotica* Fortune ex Lindl.) (Isatidis Radix in Latin, Isatis root in English, and Banlangen in Chinese) has been widely used as the remedy for fever and infection in China and other countries (Zhou and Zhang, 2013). It is well recognized for prevention and treatment effects against a variety of viral infections, such as seasonal flu (Speranza et al., 2020), severe acute respiratory syndrome (Lin et al., 2005), and H1N1 flu epidemic (Li and Tao, 2013; Luo et al., 2019). As the important ingredient in the so-called Three Drugs and Three Prescriptions, the dried root of *Isatis tinctoria* has also been playing an active role in fighting against the novel corona virus disease 2019 (COVID-19) (Li et al., 2020b; Li and Xu, 2021). Till now, more than 300 components have been isolated from the root of *Isatis indigotica*, including alkaloids, sulfur-containing compounds, phenylpropanoids, amino acids, nucleosides, organic acids and esters, flavonoids, quinones, terpenes, sterols, saccharides, aromatics, peptides, alcohols, aldehydes and ketones, nitriles, and sphingolipids. Although chemical composition and pharmacological activities of phytochemicals in the dried root of *Isatis tinctoria* have been extensively investigated by Lin et al. (2005); Speranza et al. (2020), the analysis of their spatial distribution in tissue has not yet been done.

Unraveling the tissue-specific localization of molecules in medicinal herbs can provide straightforward clues to understand their biological functions. Technologies for this aim face challenges and are still under development. Conventional investigations have enabled the comprehensive chemical profiling of metabolites from the dried root of *Isatis tinctoria*, applying separation and identification techniques such as high-performance liquid chromatography (HPLC) (Zou et al., 2005), ultra-performance liquid chromatography (UPLC) (Shi et al., 2012), and ultra-performance liquid chromatography–quadrupole–time-of-flight mass spectrometry (UPLC-Q-TOF/MS) (Guo et al., 2020a). However, the microscopic localizations of components in samples are largely ignored during the homogenization and purification process.

By directly detecting the ion beams of components on a sample surface, mass spectrometry imaging (MSI) can achieve the chemical distribution information. With the aid of the optical microscope, MSI can link morphological features with chemical profiling, thus providing untargeted, label-free, and multiplexed approach for molecular imaging. In recent years, it has become a fascinating tool for capturing the spatial accumulation and localization of metabolites in plants (Shimma and Sagawa, 2019; Li et al., 2020a; Li et al., 2020c), but only a few studies had been conducted on the dried herbal materials (Ng et al., 2007; Wu et al., 2007; Yi et al., 2012; Liang et al., 2014a; Liang et al., 2014b), which are extremely difficult to sectioning.

In the present study, distribution of phytochemicals in the dried root of *Isatis tinctoria* was revealed by microscopic mass spectrometry imaging, using atmospheric pressure–matrix-assisted laser desorption/ionization (AP-MALDI) combined with ion trap–time-of-flight mass spectrometry (IT-TOF/MS). In particular, the slice preparation method directly associated with the quality of the mass spectrometry imaging (MSI) results was optimized for the dried woody sample. Numerous constituents, including alkaloids, sulfur-containing compounds, phenylpropanoids, amino acids, nucleosides, organic acids, flavonoids, terpenes, saccharides, aromatics, peptides, and sphingolipids, were comprehensively visualized in the dried root of *Isatis tinctoria* for the first time. Moreover, samples from different habitats were distinguished based on their mass spectrometry imaging and the partial least square (PLS) regression.

## Material and Methods

### Chemicals and Samples

**FIGURE 1:**
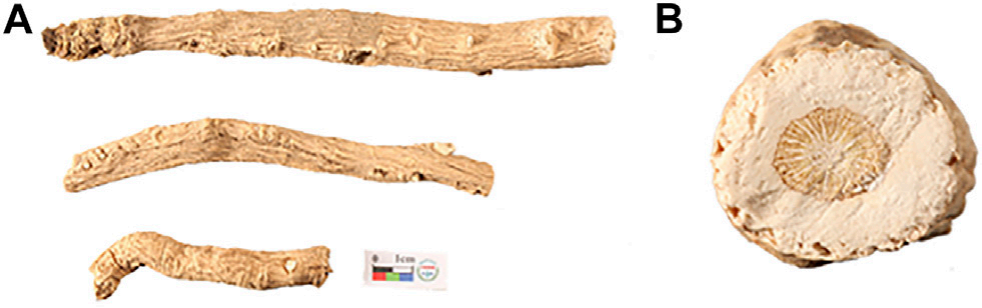
A photograph of the dried root of *Isatis indigotica* **(A)** and the magnified image of its transverse section **(B)**.

2′, 5′-Dihydroxyacetophenone (2, 5-DHAP), 2, 5-dihydroxybenzoic acid (DHB), α-cyano-4-hydroxycinnamic acid (CHCA), 1, 5-naphthalenediamine (1, 5-DAN), 9-aminoacridine (9-AA), 1, 8-bis(tetramethylguanidino)naphthalene (TMGN), 1, 2-bis (trimethoxysilyl)ethane (BTME), gelatin, formic acid (LC-MS grade), ethanol (HPLC grade), and methanol (LC-MS grade) were purchased from Sigma-Aldrich (St. Louis, MO, United States ). De-ionized water was purified by a Milli-Q system (Millipore, Bedford, MA, United States). Optimum cutting temperature (OCT) compound was purchased from Leica (Nussloch, Germany). The samples of the dried root of *Isatis tinctoria* were collected from four main habitats in China, Gansu, Heilongjiang, Xinjiang, and Neimenggu. The sample collection information could be found in Table 1, and the typical macroscopic images of the herb and its transverse section are shown in Figure 1. All samples were authenticated by Associate Professor Shuai Kang, who is in charge of the Traditional Chinese Medicine Herbarium, National Institutes for Food and Drug Control. The voucher specimens were deposited in National Institutes for Food and Drug Control (NIFDC), Beijing, China.

**TABLE 1:** Sample collection information of the dried root of *Isatis indigotica*.

Sample no	Habitat	Collection time
GS1	Gansu	Sep., 2019
GS2	Gansu	Sep., 2019
GS3	Gansu	Sep., 2019
HLJ1	Heilongjiang	Oct., 2019
HLJ2	Heilongjiang	Oct., 2019
HLJ3	Heilongjiang	Nov., 2019
XJ1	Xinjiang	Sep., 2019
XJ2	Xinjiang	Oct., 2019
XJ3	Xinjiang	Oct., 2019
NMG1	Neimengu	Nov., 2019
NMG2	Neimengu	Oct., 2019
NMG3	Neimengu	Oct., 2019

### Slice Preparation and Optical Imaging

A piece 1 cm in length was cut from the dried root of *Isatis tinctoria* using a blade, and then embedded in 0.1 g/ml gelatin solution before freezing at −20°C. The frozen sample embedded with gelatin was axially fixed on a cryomicrotome (Leica CM 1950, Nussloch, Germany) by OCT compound carefully to avoid contamination on the surface of the sample. The gelatin on the top was peeled off to expose the surface of the cross section of the root before one side of the double-sided conductive tape (3M, St. Paul, MN, United States) was adhered to the root surface. Then the tissue was sectioned into a 40-μm slice with the tape at −18°C. Finally, the slice was fixed carefully on an indium tin oxide (ITO)-coated glass slide (Matsunami Glass, Osaka, Japan) with another side of the tape. Before matrix coating, the optical image of the tissue was captured by a charge-coupled device (CCD) camera of the optical microscope embedded to the imaging mass microscope system (Shimadzu iMScope, Kyoto, Japan).

### Matrix Application

After optimization, 2′, 5′-dihydroxyacetophenone (2, 5-DHAP) and 1, 5-naphthalenediamine (1, 5-DAN) were chosen as the matrix for positive and negative ion detection, respectively, and were applied in the mode of spraying. The matrix solution of 2′, 5′-dihydroxyacetophenone (2, 5-DHAP) was prepared at a concentration of 10 mg/ml in methanol and water (all containing 0.1% formic acid) at a ratio of 8:2, and 1, 5-naphthalenediamine (1, 5-DAN) was prepared as a saturated solution in ethanol–water (70:30). For spraying, 1 ml of matrix solution was added to the cavity of the handle airbrush. Then the solution was sprayed on the sample surface using the airbrush, keeping a distance at about 10 cm. For each glass, the airbrushing was repeated 10 cycles every 60 s. Finally, the sprayed glass slide was kept in the fume hood for 5 min to vaporize the solvent.

### Microscopic Mass Spectrometry Imaging

Microscopic mass spectrometry imaging of the tissue was performed using the iMScope instrument (Shimadzu, Kyoto, Japan) equipped with an optical microscope, an atmospheric pressure chamber for matrix-assisted laser desorption/ionization (AP-MALDI) source, and an ion trap–time-of-flight mass spectrometer. The acquisition region was defined with the help of the optical microscope, and the tissue was irradiated with a diode-pumped 355 nm Nd:YAG laser with 5 ns pulse width. The laser diameter was 80 μm, and the data were collected at an interval of 80 μm. The tissue surface was laser-irradiated with 100 shots (1,000 Hz repetition rate) for each pixel. All the data were acquired in the positive and negative modes with sample voltage of 3.5 and 3.0 kV, respectively. The mass ranges were *m*/*z* 100–500 and *m*/*z* 500–1,000, while the detector voltage was 1.97 kV for all the samples. Three repetitions for each sample were performed, and two slices were prepared for each repetition in order to measure the positive and negative ions separately.

### Data Analysis

Mass image reconstruction and data analysis were performed using IMAGEREVEAL™ MS (Shimadzu, Kyoto, Japan). All images were reconstructed by linear smoothing and displayed in absolute intensity after total ion current (TIC) normalization. Statistical analysis including principal component analysis (PCA) and partial least squares (PLS) regression was carried out by IMAGEREVEAL™ MS for differentiation of samples from different habitats.

## Results

### Phytochemical Profiles of the Dried Root of *Isatis tinctoria*

**FIGURE 2:**
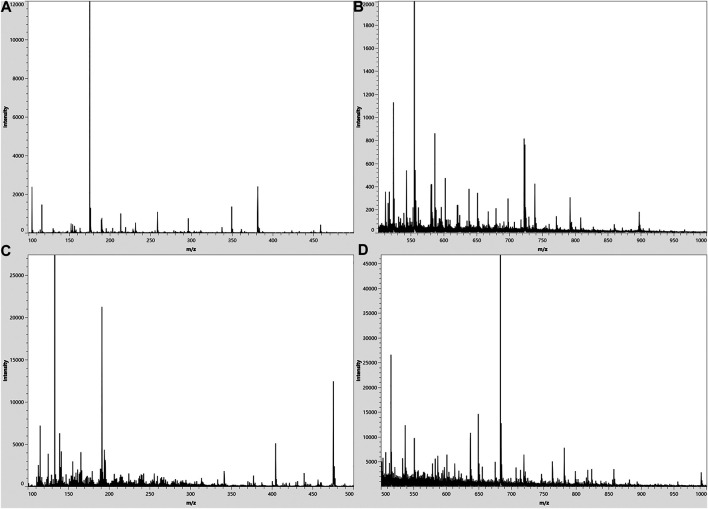
Typical overall average mass spectra acquired from a cross section of the dried root of *Isatis indigotica* by matrix-assisted laser desorption and ion trap–time-of-flight (MALDI-IT-TOF) mass spectrometry imaging (MSI) in the spectral ranges of *m*/*z* 100–500 in a positive mode **(A)**, *m*/*z* 500–1,000 in a positive mode **(B)**, *m*/*z* 100–500 in a negative mode **(C)**, and *m*/*z* 500–1,000 in a negative mode **(D)**.

Figure 2 showed the typical overall average mass spectra of the dried root of *Isatis tinctoria* gained by matrix-assisted laser desorption/ionization and ion trap–time-of-flight (MALDI-IT-TOF) mass spectrometry imaging (MSI) under positive and negative/ionization modes. Positive ions were mainly detected in the mass range of *m*/*z* 100–400 and *m*/*z* 400–800, while negative ions were mainly observed in the mass range of *m*/*z* 100–200, *m*/*z* 400–500, and *m*/*z* 400–700. The putative identification of the components was based on the accurate mass-to-charge ratio with reference to the isotopic peak, the reference standards, and/or the literatures and data bases. As could be seen from Table 2, the detected phytochemicals belong to a wide range of chemical compound classes such as alkaloids, sulfur-containing compounds, phenylpropanoids, amino acids, nucleosides, organic acids, flavonoids, terpenes, saccharides, aromatics, peptides, and sphingolipids. In the positive ion mode, the detected ions were prominently in the protonated adduct form of all amino acids, most of the alkaloids, some of the phenylpropanoids and the nucleosides, majority of the aromatics, minority of the sulfur-containing compounds, several sphingolipids, as well as organic acids with basic group. Also sodium or potassium adducts of some nucleosides and a few alkaloids, peptides, and sulfur-containing compounds were found. In the negative ion mode, majority of the sulfur-containing compounds and the organic acids, some of the phenylpropanoids, minority of the aromatics, a few saccharides, flavonoids, nucleosides, as well as alkaloids with acid group were readily detected as [M−H]^−^ form of ions.

**TABLE 2:** Assignment of ions observed in the matrix-assisted laser desorption and ion trap–time-of-flight (MALDI-IT-TOF) mass spectrometry imaging (MSI) of the dried root of *Isatis indigotica* with references regarding shown compounds.

No	Compound	Chemical class	Ion formula	Theoretical m/z	Observed m/z	Mass accuracy (ppm)	Refs
1	*r*-Aminobutyric acid	Amino acids	C_4_H_9_NO_2_+H	104.0712	104.0709	2.9	Pan, (2014)
2	Choline	Alkaloids	C_5_H_14_NO^+^	104.1075	104.1066	8.6	The Metabolomics Innovation Center, (2020)
3	Proline	Amino acids	C_5_H_9_NO_2_+H	116.0712	116.0709	2.6	Wu et al. (1997)
4	Valine	Amino acids	C_5_H_11_NO_2_+H	118.0869	118.0865	3.4	Chen et al. (2012)
5	Leucine/isoleucine	Amino acids	C_6_H_13_NO_2_+H	132.1025	132.1018	5.3	Zhao, (2015)
6	Adenine	Nucleosides	C_5_H_5_N_5_+H	136.0624	136.0613	8.1	Chen et al. (2012)
7	Aminobenzoic acid	Organic acids	C_7_H_7_NO_2_+H	138.0556	138.0545	8.0	Wang et al. (2013)
8	4-(2-Hydroxyethyl) phenol	Aromatics	C_8_H_10_O_2_+H	139.0760	139.0773	9.3	Wang et al. (2013)
9	Hexyl isothiocyanate	Alkaloids	C_7_H_13_NS + H	144.0848	144.0853	3.5	Condurso et al. (2006)
10	3-Formylindole	Alkaloids	C_9_H_7_NO + H	146.0607	146.0614	4.8	Zhao, (2015)
11	Glutamine	Alkaloids	C_5_H_10_N_2_O_3_+H	147.0770	147.0758	8.2	Pan, (2014)
12	Lysine	Amino acids	C_6_H_14_N_2_O_2_+H	147.1154	147.1132	1.4	Pan, (2014)
13	Uracil	Nucleosides	C_4_H_4_N_2_O_2_+K	151.1256	151.1243	8.6	Pan et al. (2013)
14	Guanine	Nucleosides	C_5_H_5_N_5_O + H	152.0573	152.0588	9.9	Pan et al. (2013)
15	Dopamine	Alkaloids	C_8_H_11_NO_2_+H	154.0791	154.0790	0.6	HighChem LLC, (2020)
16	Oxindole	Alkaloids	C_8_H_7_NO + Na	156.0426	156.0416	6.4	Wang et al. (2013)
17	Histidine	Amino acids	C_6_H_9_N_3_O_2_+H	156.0774	156.0786	7.7	Pan, (2014)
18	Hypoxanthine	Nucleosides	C_5_H_4_N_4_O + Na	159.0283	159.0275	5.0	Xiao et al. (2003)
19	3-Indoleformic acid	Alkaloids	C_9_H_7_NO_2_+H	162.0556	162.0553	1.9	Yang et al. (2014b)
20	Phenylalanine	Amino acids	C_9_H_11_NO_2_+H	166.0869	166.0853	9.6	Pan, (2014)
21	Acetovanillone	Aromatics	C_9_H_10_O_3_+H	167.0709	167.0709	0.0	Wang et al. (2013)
22	Isatin	Alkaloids	C_8_H_5_NO_2_+Na	170.0218	170.0221	1.8	Zou and Koh, (2017)
23	2,5-Dihydroxyindole	Alkaloids	C_8_H_7_NO_2_+Na	172.0375	172.0388	7.6	Li, (2010a)
24	Arginine	Amino acids	C_6_H_14_N_4_O_2_+H	175.1196	175.1188	4.6	Zeng et al. (2010)
25	3-Indoleacetonitrile	Alkaloids	C_10_H_8_N_2_+Na	179.0585	179.0592	3.9	Wang et al. (2013)
26	Tyrosine	Amino acids	C_9_H_11_NO_3_+H	182.0818	182.0800	9.9	Pan, (2014)
27	Dihydroconiferyl alcohol	Phenylpropanoids	C_10_H_14_O_3_+H	183.1022	183.1036	7.6	Wang et al. (2013)
28	4-Hydroxyindole-3-carboxaldehyde	Alkaloids	C_9_H_7_NO_2_+Na	184.0375	184.0368	3.8	Li et al. (2010b)
29	2,4(1H,3H)-Quinazolinedione	Alkaloids	C_8_H_6_N_2_O_2_+Na	185.0327	185.0326	0.5	Wang and Liu, 2008
30	Deoxyvasicinone	Alkaloids	C_11_H_10_N_2_O + H	187.0872	187.0864	4.3	Zhao, 2015
31	1-Methoxy-3-indoleformic acid	Alkaloids	C_10_H_9_NO_3_+H	192.0661	192.0648	6.8	Yang et al. (2014b)
32	(1′*R*,2′*R*,3′*S*,4′*R*)-1,2,4-triazole	Nucleosides	C_7_H_11_N_3_O_4_+H	202.0829	202.0841	5.9	Pan, (2014)
33	Tryptophan	Amino acids	C_11_H_12_N_2_O_2_+H	205.0978	205.0959	9.3	Pan, (2014)
54	*L*-targinine	Peptides	C_7_H_16_N_4_O_2_+Na	211.1165	211.1150	7.1	The Metabolomics Innovation Center, (2020)
35	(−)-(*R*)-2-(4-Hydroxy-2-oxoindolin-3-yl)-acetamide	Alkaloids	C_10_H_10_N_2_O_3_+Na	229.0589	229.0576	5.7	Chen et al. (2012)
36	2′-Deoxyuridine	Nucleosides	C_9_H_12_N_2_O_5_+H	229.0825	229.0842	7.4	Pan, (2014)
37	(+)-(*S*)-2-(3-Hydroxy-4-methoxy-2-oxoindolin-3-yl)-acetamide	Alkaloids	C_11_H_12_N_2_O_4_+H	237.0876	237.0899	9.7	Chen et al. (2012)
38	2′-Deoxycytidine	Nucleosides	C_9_H_13_N_3_O_4_+Na	250.0804	250.0824	8.0	Pan, (2014)
39	(*S*)-(−)-Spirobrassinin	Sulfur-containing compounds	C_11_H_10_N_2_OS_2_+H	251.0314	251.0335	8.4	Zhang et al. (2020b)
40	Pyrraline	Amino acids	C_12_H_18_N_2_O_4_+H	255.1339	255.1332	2.7	The Metabolomics Innovation Center, (2020)
41	Indiforine C	Alkaloids	C_12_H_14_N_2_O_3_+Na	257.0902	257.0918	6.2	Liu et al. (2018b)
42	Indirubin/indigotin	Alkaloids	C_16_H_10_N_2_O_2_+H	211.0813	211.0821	3.0	Chen et al. (2012)
43	Thymidine	Nucleosides	C_10_H_14_N_2_O_5_+Na	265.0801	265.0824	8.7	Pan, (2014)
44	Adenosine	Nucleosides	C_10_H_13_N_5_O_4_+H	268.1047	268.1011	6.0	Zhao, (2015)
45	Inosine	Nucleosides	C_10_H_12_N_4_O_5_+H	269.0887	269.0905	6.7	Pan et al. (2013)
46	Tryptanthrin	Alkaloids	C_15_H_8_N_2_O_2_+Na	271.0484	271.0488	1.5	Chen et al. (2012)
47	2′-*O*-Methyladenosine	Nucleosides	C_11_H_15_N_5_O_4_+H	282.1203	282.1221	6.4	Zhang et al. (2019a)
48	Indican	Alkaloids	C_14_H_17_NO_6_+H	296.0925	296.0935	3.4	Zou and Koh, (2017)
49	Guanosine	Nucleosides	C_10_H_13_N_5_O_5_+Na	306.0815	306.0824	2.9	Xiao et al, (2014)
50	(−)-(2′*S*)-Isatiscaloids E/(+)-(2′*R*)-isatiscaloids E	Alkaloids	C_14_H_19_N_3_O_5_+H	310.1404	310.1428	7.7	Wang et al. (2009)
51	Isatiscaloids A	Alkaloids	C_15_H_22_N_2_O_5_+H	311.1608	311.1627	6.1	Wang et al. (2009)
52	Indiforine F	Alkaloids	C_14_H_18_N_2_O_5_+Na	317.1114	317.1138	7.6	Liu et al. (2018b)
53	Evofolin-B	Phenylpropanoids	C_17_H_18_O_6_+H	319.1182	319.1160	6.9	Wang et al. (2013)
54	Isatiscaloids B	Alkaloids	C_16_H_20_N_2_O_5_+H	321.1451	321.1458	2.2	Wang et al. (2013)
55	Adenosine-3′,5′-cyclic monophosphate	Nucleosides	C_10_H_12_N_5_O_6_P + H	330.0604	330.0618	4.2	Pan, (2014)
56	Indole-3-acetonitrile-6-*O*-β-*D*-glucopyranoside	Alkaloids	C_16_H_18_N_2_O_6_+H	335.1244	335.1240	1.2	He et al. (2006a)
57	Isatisindigoticanine K	Alkaloids	C_19_H_13_N_3_O_2_+Na	338.0906	338.0927	6.2	Zhang et al. (2020c)
58	Coniferin	Phenylpropanoids	C_16_H_22_O_8_+H	543.1394	543.1371	6.7	Zhang et al. (2019a)
59	Isaindigodione	Alkaloids	C_18_H_18_N_2_O_4_+Na	549.1165	549.1133	9.2	Xiao et al. (2014)
60	Cyclo (*L*-Phe–*L*-Tyr)	Peptides	C_18_H_18_N_2_O_3_+Na	549.2300	549.2320	5.7	Wang et al. (2013)
61	Indole-3-acetonitrile-2-*S*-*β*-*D*-glucopyranoside	Sulfur-containing compounds	C_16_H_18_N_2_O_5_S + H	351.1015	351.1005	2.8	Yang et al. (2014b)
62	Qingdainone	Alkaloids	C_23_H_13_N_3_O_2_+ H	364.1087	364.1079	2.2	He et al. (2006a)
11	Isatindigotindoline C	Alkaloids	C_23_H_21_N_3_O_4_+H	404.1611	404.1602	2.2	Liu et al. (2018a)
64	Isatisindigoticanine A	Alkaloids	C_22_H_18_N_2_O_6_+H	407.1244	407.1215	7.1	Zhang et al. (2019b)
65	Isatindigobisindoloside G	Sulfur-containing compounds	C_24_H_21_N_3_O_5_S + H	455.1278	455.1238	8.8	Zhang et al. (2020a)
66	3-[2′-(5′-hydroxymethyl)furyl]-1(2H)-isoquinolinone-7-*O*-β-*D*-glucoside	Alkaloids	C_20_H_21_NO_9_+K	458.2199	458.2167	7.0	He et al. (2006b)
67	Isatisindigoticanine I	Sulfur-containing compounds	C_24_H_21_N_3_O_5_S + H	464.1281	464.1245	7.8	Zhang et al. (2020a)
68	Isatindigoside F	Sulfur-containing compounds	C_25_H_23_N_3_O_5_S-H	476.1279	476.1258	4.4	Zhang et al. (2020a)
69	Isatigotindolediosides B	Alkaloids	C_20_H_25_NO_11_ + Na	478.1326	478.1307	4.0	Meng et al. (2017b)
70	Isatigotindolediosides A	Alkaloids	C_21_H_27_NO_12_ + H	486.1612	486.1579	6.8	Meng et al. (2017b)
71	Isatindigoside J	Alkaloids	C_25_H_27_N_3_O_8_+H	498.1877	498.1892	3.0	Zhang et al. (2020c)
72	Isatithioetherin A/isatithioetherin B	Sulfur-containing compounds	C_20_H_26_N_4_O_4_S_3_+Na	505.1014	505.1058	8.7	Guo et al. (2020b)
73	Bisindigotin	Alkaloids	C_32_H_18_N_4_O_2_+Na	513.1328	513.1278	9.7	Wei et al. (2005)
74	Isatigotindolediosides D	Alkaloids	C_22_H_28_N_2_O_13_ + H	529.1670	529.1111	7.4	Meng et al. (2017b)
75	Isatithioetherin C/isatithioetherin E	Sulfur-containing compounds	C_20_H_26_N_4_O_4_S_4_+Na	537.0735	537.0784	9.1	Guo et al. (2020b)
76	(+)-(7*R*,7′*R*,8*S*,8′*S*)-Neo-olivil	Phenylpropanoids	C_26_H_54_O_12_ + H	539.2129	539.2143	2.6	Kikuchiand Kikuchi, (2005)
77	(2*S*,3*R*)-3-Hydroxymethyl-*N*-(2′-hydroxynonacosanoyl)-trideca-9*E*-sphingenine	Sphingolipids	C_43_H_85_NO_5_+H	696.6507	696.6565	8.3	Li et al. (2007)
78	1-O-β-D-Glucopyranosyl-(2*S*,3*R*)-*N*-(2′-hydroxyhe xacosanoyl)-octadeca-11*E*-sphingenine	Sphingolipids	C_50_H_97_NO_9_+H	856.7242	856.7292	5.8	Sun et al. (2009)
79	Propanedioic acid	Organic acids	C_3_H_4_O_4_-H	103.0031	103.0040	8.7	Li, (2010)
80	Pyrocatechol	Aromatics	C_6_H_6_O_2_-H	109.0289	109.0294	4.6	Wang et al. (2013)
81	Maleic acid/fumaric acid	Organic acids	C_4_H_4_O_4_-H	115.0031	115.0040	7.8	Peng et al. (2005b)
82	Nicotinic acid	Organic acids	C_6_H_5_NO_2_-H	122.0241	122.0254	5.7	Li, (2010)
83	3-Methylfuran-2-carboxylic acid	Organic acids	C_6_H_6_O_3_-H	125.0238	125.0243	4.0	Zeng et al. (2010)
84	Goitrin/epigoitrin	Sulfur-containing compounds	C_5_H_7_NOS-H	128.0169	128.0162	5.5	Wang et al. (2014)
85	Malic acid	Organic acids	C_4_H_6_O_5_-H	133.0136	133.0147	8.3	Liu et al. (2010)
86	Salicylic acid	Organic acids	C_7_H_6_O_3_-H	137.0238	137.0241	2.2	Zhao, (2015)
87	Vanillin	Aromatics	C_8_H_8_O_3_-H	151.0394	151.0388	4.0	Sun et al., 2007
88	Fructose/glucose	Saccharides	C_6_H_12_O_6_-H	179.0555	179.0554	0.6	Liu et al. (2010)
89	Mannitol	Saccharides	C_6_H_14_O_6_-H	181.0711	181.0697	7.7	He et al., 2006b
90	2-Amine-4-quinlinecarboxylic acid	Alkaloids	C_10_H_8_N_2_O_2_-H	187.0507	187.0517	5.3	Pan, (2014)
91	Citric acid	Organic acids	C_6_H_8_O_7_-H	191.0191	191.0191	0.0	Liu et al. (2010)
92	Glucuronic acid	Organic acids	C_6_H_10_O_7_-H	193.0548	193.0338	5.2	Liu et al. (2010)
93	Syringic acid	Organic acids	C_9_H_10_O_5_-H	197.0461	197.0449	6.1	Wang et al. (2009)
94	Isatindosulfonic acid E	Sulfur-containing compounds	C_9_H_9_NO_3_S-H	210.0224	210.0233	4.3	Meng et al. (2017a)
95	Isatindosulfonic acid C	Sulfur-containing compounds	C_10_H_11_NO_4_S-H	240.0330	240.0318	5.0	Meng et al. (2017a)
96	Palmitic acid	Organic acids	C_16_H_32_O_2_-H	255.2323	255.2542	7.4	Kizil et al. (2009)
97	Emodin	Flavonoids	C_15_H_10_O_5_-H	269.0449	269.0450	0.4	Li, (2010)
98	Linolenic acid	Organic acids	C_18_H_30_O_2_-H	277.2167	277.2172	1.8	Kizil et al. (2009)
99	Calycosin	Flavonoids	C_16_H_12_O_5_-H	283.0606	283.0111	8.8	Wang et al. (2013)
100	Stearic acid	Organic acids	C_18_H_36_O_2_-H	283.2116	283.2646	3.5	Kizil et al. (2009)
101	Sucrose	Saccharides	C_12_H_22_O_11_-H	541.1083	541.1089	1.8	Liu et al. (2010)
102	Sinensetin	Flavonoids	C20H20O7- H	371.1130	371.1103	7.3	Li, (2010)
103	Gluconapin	Sulfur-containing compounds	C_11_H_19_NO_9_S_2_-H	372.0422	372.0429	1.9	Mohn et al. (2007)
104	Isatindigotindoloside C/Isatindigotindoloside D	Sulfur-containing compounds	C_17_H_20_N_2_O_6_S-H	379.0911	379.0938	6.6	Liu et al. (2015b)
105	Progoitrin/epiprogoitrin	Sulfur-containing compounds	C_11_H_19_NO_10_S_2_-H	388.0371	388.0355	4.1	Mohn et al. (2007)
106	Glucotropaeolin	Sulfur-containing compounds	C_14_H_19_NO_9_S_2_-H	408.0422	408.0418	1.0	Mohn et al. (2007)
107	Isovitexin	Flavonoids	C_21_H_20_O_10_-H	431.0977	431.1012	8.1	Zhao, (2015)
108	Glucobrassicin	Sulfur-containing compounds	C_16_H_19_N_2_O_9_S_2_-H	447.0531	447.0537	1.3	Guo et al. (2020a)
109	Isatindigobisindoloside A/isatindigobisindoloside B	Alkaloids	C_24_H_23_N_3_O_6_-H	448.1508	448.1554	5.8	Liu et al. (2015a)
110	Isatindigoside F	Sulfur-containing compounds	C_25_H_23_N_3_O_5_S-H	476.1279	476.1258	4.4	Zhang et al. (2020a)
111	Isatigotindolediosides F	Sulfur-containing compounds	C_21_H_27_NO_12_S-H	516.1175	516.1153	4.3	Meng et al. (2017b)
112	Isatigotindolediosides E	Sulfur-containing compounds	C_22_H_28_N_2_O_11_S-H	527.1335	527.1284	9.7	Meng et al. (2017b)
113	Isatigotindolediosides D	Sulfur-containing compounds	C_22_H_28_N_2_O_13_-H	527.1512	527.1538	4.9	Meng et al. (2017b)
114	Glucoisatisin/epiglucoisatisin	Sulfur-containing compounds	C_21_H_26_N_2_O_12_S_2_-H	561.0848	561.0830	3.2	Mohn and Hamburger, (2008)
115	Isovitexin	Flavonoids	C_21_H_20_O_10_-H	431.0977	431.1012	8.1	Zhao, (2015)
116	Linarin	Flavonoids	C_28_H_32_O_14_-H	591.1713	591.1674	6.6	Peng et al. (2005a)
117	Neohesperidin	Flavonoids	C_28_H_54_O_15_-H	609.1819	609.1801	3.0	Peng et al. (2005a)
118	Clemastanin B	Phenylpropanoids	C_32_H_44_O_16_-H	683.2550	683.2489	8.9	Yang et al. (2014a)

### Visualization of the Distribution of Phytochemicals in the Dried Root of *Isatis tinctoria*

**FIGURE 3:**
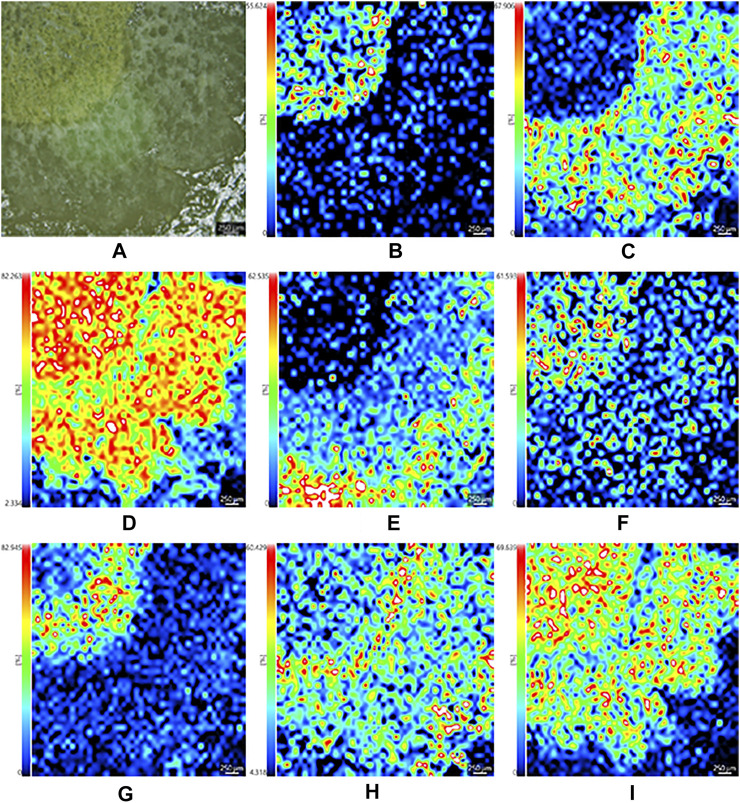
Optical image of the dried root of *Isatis indigotica* **(A)** and the mass spectrometry images of the positive ions of oxindole **(B)**, 3-[2′-(5′-hydroxymethyl)furyl]-1(2*H*)-isoquinolinone-7-O-β-D-glucoside **(C)**, coniferin **(D)**, guanine **(E)**, histidine **(F)**, proline **(G)**, arginine **(H)**, and cyclo (*L*-Phe–*L*-Tyr) **(I)**.

**FIGURE 4:**
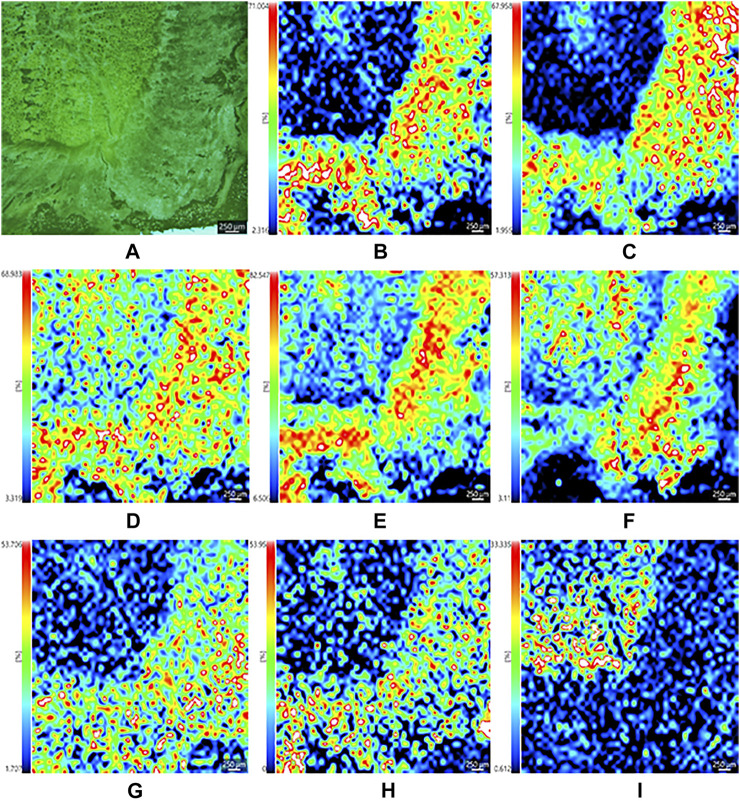
Optical image of the dried root of *Isatis indigotica* **(A)**, and the mass spectrometry images of the negative ions of isatindigoside F **(B)**, clemastanin B **(C)**, maleic acid **(D)**, malic acid **(E)**, citric acid **(F)**, sucrose **(G)**, isovitexin **(H)**, and vanillin **(I)**.

The optical images in Figures 3, 4 showed the main compartments of the cross section of the dried root of *Isatis tinctoria*: cork and cortex, phloem, cambium, and xylem as well as the distinctive spatial distribution of various kinds of characteristic constituents. The most abundant class of chemical components isolated from *Isatis tinctoria* is alkaloid. They were mostly detected as the positive ions and presented a variety of distributions. As could be seen from Figure 3B, sodium adduct of oxindole (*m*/*z* 156.0416), an indole alkaloid, was located exclusively in xylem. Another ion of *m*/*z* 458.2167 (Figure 3C) was found to have a different distribution in the specific region of phloem. This ion was assigned to the potassium adduct of 3-[2′-(5′-hydroxymethyl) furyl]-1(2*H*)-isoquinolinone-7-*O*-β-*D*-glucoside, an isoquinolinone alkaloid glycoside. Sulfur-containing compounds are characteristic secondary metabolites occurring in cruciferous plant, and they are the second abundant class of chemical components in *Isatis tinctoria*. Taking isatindigoside F (*m*/*z* 476.1258), a typical glucosinolate, as an example (Figure 4B), sulfur-containing compounds were mainly detected as the negative ions which accumulated mostly in phloem of the dried root of *Isatis tinctoria*. Dozens of phenylpropanoids are also found in *Isatis indigotica*, and they can be detected in positive or negative modes. Figure 3D and Figure 4C showed the spatial distribution of the ions of *m*/*z* 343.1371 and *m*/*z* 683.2479, which corresponded to the [M+H]^+^ and [M−H]^−^ forms of the ions of coniferin and clemastanin B, a phenylpropanoid glycoside and a lignan diglucoside, respectively. Regardless of the form of the ions, the majority of the phenylpropanoids presented the highest abundance near the lateral area of the root, including phloem, cortex, and cork. *Isatis* root produces several nucleosides, which are normally observed as positive ions. MSI results suggested that the ion of *m*/*z* 152.0588 was assigned to the protonated adduct of guanine. As noticeable in the ion, it was located almost exclusively in xylem of the root. Moreover, mass spectrometry imaging (MSI) study on the constituents of the dried root of *Isatis tinctoria* revealed the presence of a variety of organic acids. The negative ions assigned as [M−H]^−^ form of maleic acid (*m*/*z* 115.004), malic acid (*m*/*z* 133.0147, Figure 4E), and citric acid (*m*/*z* 191.0191, Figure 4F) were found at very high intensities in the area of phloem. Amino acids are widespread primary metabolites in plants, and they showed diversified distribution in the dried root of *Isatis tinctoria* in the form of the proton adducts. The protonated adduct of histidine at *m*/*z* 156.0786 (Figure 3F) was more abundant at the inner side of the cambium. On the contrary, another ion image concerned the distribution of proline at *m*/*z* 116.0709 (Figure 3G) with the highest abundance at the outer side of the cambium. Interestingly, the ion of arginine at *m*/*z* 175.1188 (Figure 3H) was located with prominent abundance in all tissues except regions of cambium, cortex, and cork. Ions based on phytochemicals belonging to minor groups in *Isatis* root like peptides, saccharides, flavonoids, and aromatics were also analyzed. It could be judged from Figure 3I that the highest concentration of a cyclic peptide named cyclo (*L*-Phe–*L*-Tyr) in the form of potassium adduct at *m*/*z* 349.2320 was in xylem and phloem. Sucrose is a nutritional ingredient that naturally occurs in many plants. Its negative ion at *m*/*z* 341.1089 (Figure 4G) was located mainly in the outer part of the root corresponding to the location of phloem, cortex, and cork. Similar distribution pattern was observed for the ion assigned to isovitexin (*m*/*z* 431.1012, Figure 4H), a representative flavone glycoside from *Isatis* root. Compounds belonging to aromatics are also isolated from *Isatis* root. This group includes vanillin, whose negative ion could be found at *m*/*z* 151.0388. In contrast to the above-discussed localization of two ions, this component accumulated at the area corresponding to xylem mainly (Figure 4I).

### Differentiation of the Habitats of the Dried Root of *Isatis tinctoria*

**FIGURE 5:**
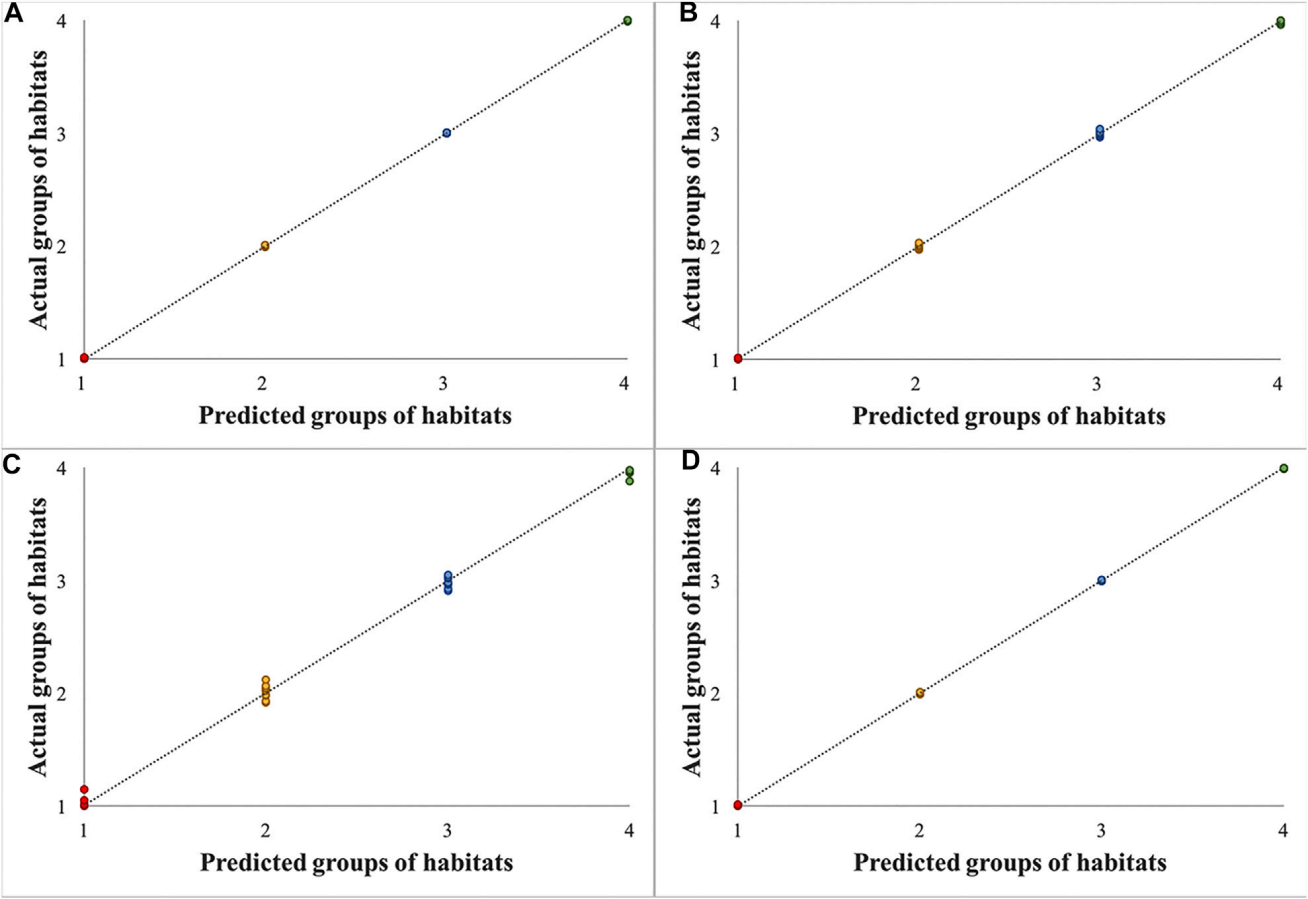
Results of partial least square (PLS) regression models for samples of the dried root of *Isatis indigotica* from four habitats based on mass spectrometry imaging (MSI) data in the spectral ranges of *m*/*z* 100–500 in a positive mode **(A)**, *m*/*z* 500–1,000 in a positive mode **(B)**, *m*/*z* 100–500 in a negative mode **(C)**, and *m*/*z* 500–1,000 in a negative mode **(D)**.

Three repetitions for twelve samples of the dried root of *Isatis tinctoria* from Gansu, Xinjiang, Heilongjiang, and Neimenggu were first classified according to their habitats as groups 1, 2, 3, and 4, respectively. Mass spectrometry imaging (MSI) data of the whole tissues within two spectral ranges (*m*/*z* 100–500 and *m*/*z* 500–1,000) in positive and negative modes were input to establish four partial least square (PLS) regression models separately. Partial least square (PLS) regression was performed by importing the information of all detected ions to the *X*-matrix, while the actual groups of habitats were imported to the *Y*-matrix. As shown in Figure 5, good correlation between the predicted and actual groups of habitats of the dried root of *Isatis tinctoria* was found with all regression coefficients (*R* ^2^) above 0.99, which indicated an excellent discrimination ability of the mass spectrometry imaging (MSI) method.

## Discussion

In this study, iMScope, the optical microscope, in combination with the atmospheric pressure–matrix-assisted laser desorption/ionization (AP-MALDI) and the ion trap–time-of-flight mass spectrometry (IT-TOF/MS), was first applied to visually clarify the distribution of phytochemicals in the dried root of *Isatis tinctoria*. Nowadays, there have been increasing reports on the applications of mass spectrometry imaging (MSI) in the investigation of animal or human tissues (Wu et al., 2019), but with less focus on plants (Davison et al., 2019; Sun et al., 2020; Zhang et al., 2020d), not to mention traditional Chinese medicine (TCM). In addition, most of the research works were performed with the fresh herbs (Fowble et al., 2017; Lange et al., 2017; Freitas et al., 2019) due to the obstacles in the sectioning of the dried material. To reveal the spatial localization of phytochemicals in traditional Chinese medicine (TCM) in the form of actually sold in the market and used in clinic, the dried root of *Isatis tinctoria* was chosen as the imaging subject. As expected, the cryosectioning of the hard and fragile woody root presented great challenges.

First, the thickness of the tissue was optimized in the range from 20 to 80 μm. It was apparent that the thicker the slice, the more integrated the tissue, but thinner slice posed more sensitive detection of ions. Taking a comprehensive consideration of the integrity of the tissue and the quality of the mass data, a thickness of 40 μm was selected. Next, the temperature for cryosectioning of the frozen tissue was assessed in the range of −12–−22°C. It was found that the section would rupture when the temperature was too low, while when the temperature was too high, the section would wrinkle. Numerous trials indicated that satisfactory result could be obtained with a temperature of −18°C, which was coincident with the reported optimum cryosectioning parameter for the roots of *Panax* genus (Wang et al., 2016). Since the thin slice of the dried root of *Isatis tinctoria* was easy to fall off from the glass slide, a double-sided adhesive tape was utilized. To better avoid tissue break and movement during the sectioning, the sample surface was adhered by one side of the tape before cutting.

Subsequently, several matrices were evaluated as follows: 2′, 5′-dihydroxyacetophenone (2, 5-DHAP), 2, 5-dihydroxybenzoic acid (DHB), α-cyano-4-hydroxycinnamic acid (CHCA), and 1, 5-naphthalenediamine (1, 5-DAN) for the positive ion mode, and 1, 5-naphthalenediamine (1, 5-DAN), 9-aminoacridine (9-AA), 1, 8-bis(tetramethylguanidino) naphthalene (TMGN), and 1, 2-bis(trimethoxysilyl)ethane (BTME) for the negative ion mode. Briefly, comprehensive detection of molecules was achieved when using 2′, 5′-dihydroxyacetophenone (2, 5-DHAP) and 1, 5-naphthalenediamine (1, 5-DAN) as the matrixes in positive and negative ion modes, respectively. It was unexpected that DHB, the regular matric used in MALDI MSI of small molecules in plants, was not the most fitted matric for the dried root of *Isatis tinctoria.* In addition, two matrix-coating modes, air-assisted spraying and sublimation, were compared, and the results indicated that spraying presented stronger signal intensity and miner analyte delocalization.

As a result, 118 ions in the dried root of *Isatis tinctoria* were assigned as 10 classes of components including some bioactive molecules. Not surprisingly, the majority of the identified phytochemicals belonged to alkaloids and sulfur-containing compounds. The second most detected components were nucleosides, organic acids, and amino acids. A few aromatics, flavonoids, phenylpropanoids, saccharides, peptides, and sphingolipids were also found. On the contrary, esters, quinones, terpenes, sterols, alcohols, aldehydes, ketones, and nitriles from *Isatis indigotica* were not detected by mass spectrometry imaging (MSI) this time. Like fructose and glucose, several alkaloids, sulfur-containing compounds, amino acids, and saccharides were grouped together in Table 2 since they hold isomeric relationship and could not be differentiated by their exact mass. Hence, further work should be done for the separation of the detected isomers. The presence of most of the identified components was previously found in *Isatis indigotica* except choline, dopamine, *L*-targinine, and pyrraline, which might be caused by the loss during the extraction and separation process in routine methods. Therefore, the matrix-assisted laser desorption/ionization and ion trap–time-of-flight (MALDI-IT-TOF) mass spectrometry imaging (MSI) in a single run covered not only the natural products that were commonly detected but also less reported molecules, illustrating the high throughput and high sensitivity of the method. It was also delighted to find that a couple of important bioactive molecules (references could be seen in Table 2) from *Isatis indigotica*, such as uracil, adenine, hypoxanthine, 4(3*H*)-quinazolinone, deoxyvasicinone, 2,4(1*H*,3*H*)-quinazolinedione, isalexin, guanine, indirubin, and indigotin, were identified in an untargeted, label-free, and multiplexed way without extraction or isolation. Among them, uracil, adenine, guanine, indirubin, and indigotin are often used as the chemical markers for authentication of the root of *Isatis indigotica*, and they were detected in all the investigated samples. Using mass spectrometry imaging, the herb could be identified in a rapid way with multiple indexes, comparing to routine TLC or HPLC methods.

**FIGURE 6:**
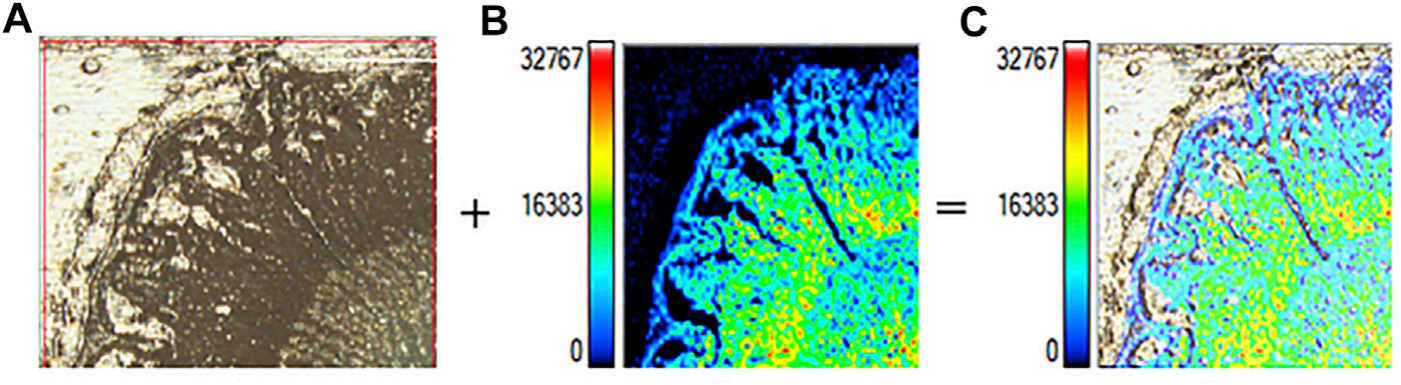
Optical image **(A)**, mass spectrometry image **(B)**, and overlay image **(C)** of the dried root of *Isatis indigotica*.

As indicated in Figure 6, the optical microscope embedded in the iMScope made it possible to acquire optical images and ion distribution images in the same instrument. The convenient assessment allowed for an unprecedented visualization of the spatial distribution of phytochemicals in the dried root of *Isatis indigotica*. Consequently, the localization and spatial information of some molecules in the root tissue were elucidated, which were related to the botanical structure of the herb. In all, a majority of the phytochemicals were shown to be more abundant in phloem, the nutrition-storing tissue of *Isatis indigotica*, than in xylem, the principle water-conducting tissue. Nevertheless, the signals for some identified constituents were considerably lower, and their mass spectrometry imaging (MSI) localization was therefore not so distinctive. Comparing with the LC-MS analysis after laser microdissection, microscopic mass spectrometry imaging using matrix-assisted laser desorption can reveal the spatial distribution of compounds in a more precise and direct way. Besides, segmentation and dissection might bring uncontrollable pollution, compound migration, or denaturation. However, the differentiation of isomers and absolute quantitation were not available by MALDI-MSI currently.

Finally, based on the ion images, data were collected from the whole tissue and analyzed by partial least squares (PLSs), and the dried root of *Isatis indigotica* from four habitats was differentiated unambiguously. Spectra collected from the whole tissue, the outer part of the tissue (cork, cortex, and phloem), and the inner part of the tissue (cambium and xylem) were also inputted for principal component analysis (PCA). However, no distinct cluster was observed for samples from different habitats.

Among other possibilities, the results from this study can be applied to increase the extraction yield of a given active component in *Isatis indigotica*, which is promising in research fields, such as pharmaceutical applications and industrial production. Moreover, the location of specific metabolites is helpful to improve the understanding of the relationship between compound distribution and plant structure as well as function. Combining with the chemometric method, mass spectrometry imaging (MSI) provides a simple and rapid approach for distinguishing habitats of traditional Chinese medicine (TCM) and exploring the environmental effects of plant growth. Finally, similar studies on other traditional Chinese medicine (TCM) are underway in our laboratory.

## Data Availability

The raw data supporting the conclusion of this article will be made available by the authors, without undue reservation, to any qualified researcher.
